# Development of insulated isothermal PCR for rapid on-site malaria detection

**DOI:** 10.1186/s12936-016-1183-z

**Published:** 2016-03-01

**Authors:** Kek Heng Chua, Ping Chin Lee, Hwa Chia Chai

**Affiliations:** Department of Biomedical Science, Faculty of Medicine, University of Malaya, 50603 Kuala Lumpur, Malaysia; School of Science and Technology, Universiti Malaysia Sabah, 88400 Kota Kinabalu, Sabah Malaysia

**Keywords:** *Plasmodium* species, Diagnostic test, Insulated isothermal polymerase chain reaction (iiPCR), Endemic rural areas, POCKIT™

## Abstract

**Background:**

Detection of *Plasmodium* spp. is sometimes inconvenient especially in rural areas that are distant from a laboratory. In this study a portable diagnostic test of *Plasmodium* spp. was developed using insulated isothermal polymerase chain reaction (iiPCR) as an alternative approach to improve this situation.

**Methods:**

A pair of universal primers and probe were designed to amplify and detect gene encoding 18S small sub-unit rRNA of *Plasmodium* spp using iiPCR method in a portable device, POCKIT™. The efficiency and detection limit of the assay were evaluated using quantitative real-time polymerase chain reaction (qPCR) approach before being subjected to testing in POCKIT™. Detection results of POCKIT™ were displayed as ‘+’, ‘−’ or ‘?’ based on the fluorescence ratio after/before reaction. A total of 55 and 35 samples from malaria patients and healthy subjects, respectively, were screened to evaluate the feasibility of this newly designed iiPCR assay.

**Results:**

The iiPCR assay allowed the detection of various species of *Plasmodium*, including those infecting humans (*Plasmodium falciparum*, *P. vivax*, *P. knowlesi*, *P. malariae*, *P. ovale*), monkeys, birds, and rodents. Efficiency of the assay achieved 96.9 % while the lower detection limit was ≥100 copies of plasmodial DNA. Specificity of the assay was assured as it could not detect human, bacterial and other parasitic DNA. Among the 55 clinical samples tested, 47 (85.4 %) of them were detected as positive by POCKIT™. Four (7.3 %) samples with fluorescence ratio after/before reaction of <1.2 were reported as negative while another four (7.3 %) were ambiguously detected as they had fluorescence ratios between 1.2 and 1.3. The fluorescence ratio was not found to be associated with the copy number of plasmodial DNA. This approach can only be considered as a qualitative method.

**Conclusions:**

The portable iiPCR system may serve as an alternative approach for preliminary screening of malaria in endemic rural areas. The system may also be useful for detecting animal malaria in the field. Although it is not as quantitative as qPCR method, it is comparatively fast and easy to handle. It is believed that the POCKIT-iiPCR assay is able to achieve 100 % sensitivity if increased amount of DNA from each sample is used. The iiPCR assay can also be upgraded in future to detect multiple *Plasmodium* spp. at the same time by designing the specific primers and probes.

## Background

Malaria is a global health issue and presently endemic in 97 countries [1]. In 2013, an estimated 198 million cases of malaria occurred worldwide with 584,000 deaths. Africa’s endemic countries have most cases (80 %) and deaths (90 %).

The majority of malaria cases are reported from rural areas. Socio-economic factors related to poverty, low health consciousness and disease prevention, and poor infrastructure and transport contribute to a higher prevalence rate of malaria in rural areas compared to urban areas [2]. All these factors hinder early treatment of the disease, and prompt the development of diagnostic methods that are easily accessible and usable without delay or the need to travel or transport patient samples to laboratories, which can take hours or days to reach. Thus, this study aimed to develop a portable, user-friendly diagnostic method that can be hand-carried into endemic rural areas.

Insulated isothermal polymerase chain reaction (iiPCR) is established based on Rayleigh-Bénard convection method, which can amplify nucleic acids into significant amounts within 30 min in a simple heating device [3–5]. It is a PCR assay whereby the copper ring attached to the bottom of a special polycarbonate capillary tube (R-tube™) is heated isothermally by the device and the PCR can occur when reagents travel through temperature gradient zones created by thermal convection in a tube [6]. Integration of fluorescent hydrolysis probe technology into iiPCR further upgrades its usefulness as detection results can be displayed directly on the device [5]. The device is now commercially available and is named POCKIT™ nucleic acid analyzer (GeneReach, Taichung City, Taiwan). The platform allows iiPCR or reverse transcription-iiPCR, and fluorescence signal detection and data interpretation upon completion of reaction within 1 h. One to eight reactions can be carried out concurrently in one run at the present setting and the device is a closed system where R-tubes™ are used and inserted into the device. The built-in algorithms in the device calculate the signal-to-noise (S/N) ratio, which is the fluorescence after/before reaction, and subsequently display them as ‘+’, ‘−’ or ‘?’ according to default thresholds [7]. The device is able to detect two fluorescence dyes, i.e., 6-FAM™ and VIC^®^ dyes at 520 and 550 nm, respectively.

Recently, several iiPCR assays were developed for detection of pathogens, including white spot syndrome virus [5, 6] and canine distemper virus [7]. The iiPCR assay for white spot syndrome virus has also been validated to have sensitivity and specificity comparable to those of nested PCR [8]. Since the device is small and portable, it is suitable for on-site pathogen detection or fieldwork purposes. Considering all the advantages, a malaria detection assay was developed based on iiPCR approach in POCKIT™.

## Methods

### Plasmid DNA preparation

Clones of plasmids carrying recombinant gene sequence of 18S small sub-unit (SSU) rRNA for five human *Plasmodium* spp. (*Plasmodium falciparum,P. vivax*, *P. knowlesi*, *P. malariae*, *P. ovale*) were obtained from a previous study [9]. Briefly, *Escherichia coli* bearing the recombinant plasmid DNA was grown overnight at 37 °C in 10 ml Luria-Bertani (LB) broth containing 100 µg/ml ampicillin with vigorous shaking. The bacterial cells were harvested in the following morning by centrifugation at 6000×*g* for 15 min at 4 °C and subsequently subjected to plasmid isolation and purification using High Yield Plasmid Mini Kit (Yeastern Biotech, Taiwan) according to manufacturer’s instructions. The purified plasmid DNA samples were quantified at 260 nm with a spectrophotometer and then kept at −20 °C until further use.

### Primers and probe design

A pair of universal primers, Isothermo (F) and Isothermo (R), was designed based on *18S SSU rRNA* gene sequences of *Plasmodium* spp. as it contains highly conserved region. The gene sequences of *P.falciparum*, *P. knowlesi*, *P. ovale*, *P. malariae,* and *P. vivax* were retrieved from GenBank (accession number M19172.1, U83876.1, L48987.1, M54897.1, X13926.1, respectively) and aligned using Clustal Omega software (EMBL-EBI, Cambridge, UK) [10]. The primers were designed using Primer Express 3.0 (Applied Biosystems, CA, USA) based on several guidelines: amplicon length is between 70 and 150 bases; GC content is between 45 and 60 %; T_m_ of primers ranges between 56 and 60 °C; more than four Gs or Cs in a row should be avoided; repeated sequences and secondary structures formation should also be avoided; and one to three Gs or Cs should be included in the last five bases at the 3′-end of the primers. The universal primer pairs designed were searched against NCBI database using Primer-Basic Local Alignment Search Tool (Primer-BLAST) (NCBI, MD, USA) [11] to ensure the assay can amplify at least all five human *Plasmodium* spp.

A TaqMan^®^ probe (Applied Biosystems), namely Isoplasmo probe, with a FAM™ dye label on the 5′-end and minor groove binder (MGB) non-fluorescent quencher (NFQ) on the 3′-end was designed using Primer Express 3.0 (Applied Biosystems) so that it would anneal on the sequence flanked between the two primers. The probe was designed according to a few guidelines: probe length is less than 30 bases; T_m_ of probe is 10 °C higher than that of primers; GC content is kept within 40–80 %; G-residue on the 5′-end of the probe should be avoided; and a stretch of more than four Gs should be avoided. The probe sequence was again undergone NCBI database search using BLAST to ensure that it could detect at least all five human *Plasmodium* spp.

### Testing of primers and probe using qPCR

Before being subjected to iiPCR, the Isothermo primers and Isoplasmo probe were tested with quantitative real-time polymerase chain reaction (qPCR) technique using an Applied Biosystems 7500 Fast Real-Time PCR System. The recombinant plasmid DNA of *P. falciparum*, *P. vivax*, *P. knowlesi*, *P. malariae*, and *P. ovale* was used as the template. Each reaction with total volume of 10 µl consisted of 1X TaqMan^®^ Fast Universal PCR Master Mix (Applied Biosystems), 50 nM of each Isothermo primer, 80 nM of Isoplasmo probe and 1 µl of recombinant plasmid DNA of variable copy numbers. The qPCR was performed under a thermal cycling condition of: enzyme activation at 95 °C for 20 s, and 40 cycles of melting and annealing/extension at 95 °C for 2 s and 60 °C for 30 s, respectively. In order to evaluate the efficiency of the assay, standard curve was constructed with six ten-fold diluted (from 10^−4^ to 10^−9^ X) recombinant plasmid DNA (initial amount of 50 ng). The qPCR was carried out in at least triplicates. The copy number of recombinant plasmid DNA was determined using a copy number calculator software freely available on the internet (Thermo Scientific, MA, USA). The ability of the assay to detect DNA of *Plasmodium* spp in clinical samples was also tested and the copy numbers of plasmodial DNA in each clinical sample were also determined based on the standard curve.

### iiPCR method development

The iiPCR was performed in a portable device, POCKIT™ (GeneReach), with the use of special tubes namely R-tubes™ (GeneReach). The concentrations of Isothermo primers and Isoplamo probe were optimized and determined using recombinant plasmid DNA as the positive controls. The final 50-µl reaction mixture composing of 1X TaqMan^®^ Fast Universal PCR Master Mix (Applied Biosystems), 100 nM of Isothermo (F), 100 nM of Isothermo (R), 80 nM of Isoplasmo probe, and 1 µl of DNA template was used in this study. The reaction mixtures were subjected to iiPCR under default thermal condition, which took 58 min to complete. The fluorescence change for FAM dye in the reaction was detected at wavelength of 520 nm and the result was displayed in the form of +, − or ?. Raw fluorescent data and fluorescence ratio after/before reaction were also analysed.

### Evaluation of iiPCR assay

The sensitivity and detection limits of the iiPCR assay were evaluated using 10-fold diluted recombinant plasmid DNA, from 10^5^ to 1 copy number(s). Furthermore, qPCR of the 10-fold diluted recombinant plasmid DNA in the 50-µl reaction mixture was also performed using Applied Biosystems 7500 Fast Real-Time PCR System to determine the efficiency of the iiPCR assay. Both iiPCR and qPCR were performed in at least triplicates. The specificity of the assay was also examined using known DNA samples of other *Plasmodium* spp as well as those of human, bacteria (*Aeromonas hydrophila*, *A. caviae*, *A. aquariorum*, *Serratia marcescens*, *Vibrio parahaemolyticus*, *V. harveyi*) and other parasites (*Cryptosporidium parvum, Giardia duodenalis, Haemonchus contortus, Trichostrongylus colubriformis, Fasciola gigantica, Dirofilaria immitis*).

### Screening of clinical samples

A total of 55 clinical DNA samples obtained from a previous study [9] were randomly selected to validate the feasibility of iiPCR assay. The clinical samples were collected from Sabah, Malaysia with protocols in accordance with approved guidelines by the Medical Ethics Board of the University of Malaya Medical Centre (UMMC) (Ethics reference no. 709.2). A total of 35 blood DNA samples from healthy donors recruited from UMMC was also obtained from a previous study. Informed consent and consent to publish were obtained from all patients and healthy subjects. Briefly, blood films were prepared from whole blood samples collected from malaria patients and examined under microscope to determine the parasitaemia levels. An aliquot of 200 µl of each blood sample was subjected to DNA extraction using QIAamp DNA Mini Kit (Qiagen, Hilden, Germany), where protocol was according to the manufacturer’s manual. The plasmodial DNA in the clinical samples were then detected using conventional hexaplex PCR [9]. From the previous microscopic and hexaplex PCR identification, the 55 selected clinical samples consisted of 34 cases of *P. falciparum* infections, 11 cases of *P. vivax* infections and 10 cases of *P. knowlesi* infections. The association between after/before fluorescence ratios and copy numbers of the plasmodial DNA in the clinical samples was also investigated.

## Results

### Primer and probe sequences and *Plasmodium* spp

Universal primer pair, Isothermo (F) 5′-CGGAAGGGCACCACCAG-3′ and Isothermo (R) 5′-TCACCATCCAAGAAATCAAGAAAG-3′, was designed to amplify a common region in the *18S SSU rRNA* gene of *Plasmodium* spp. The amplicon size was 124 bp. Besides the five human *Plasmodium* spp. (*P. falciparum*, *P. vivax*, *P. knowlesi*, *P. malariae*, *P. ovale*), Primer-BLAST analysis showed that the primers could also amplify gene sequence of *Plasmodium* spp infecting monkeys, rodents and birds (Table 1). The designed Isoplasmo probe (FAM 5′-TCCTACTCTTGTCTTAAACTA-3′ MGB-NFQ) was able to detect all *Plasmodium* spp. amplified by the primers (Table 1).

**Table 1:** *Plasmodium* spp. that could be amplified and hybridised by the primers and probe designed for POCKIT-iiPCR assay

*Plasmodium* spp.	Host
*P. falciparum*	Humans
*P. vivax*	Humans
*P. ovale*	Humans
*P. malariae*	Humans, monkeys
*P. knowlesi*	Humans, monkeys
*P. inui*	Monkeys
*P. fieldi*	Monkeys
*P. coatneyi*	Monkeys, humans
*P. cynomolgi*	Monkeys, humans
*P. fragile*	Monkeys
*P. simiovale*	Monkeys
*P. simium*	Monkeys, humans
*P. gonderi*	Monkeys
*P. hylobati*	Monkeys
*P. brasilianum*	Monkeys
*P. yoelii*	Rodents
*P. berghei*	Rodents
*P. chabaudi*	Rodents
*P. vinckei*	Rodents
*P. juxtanucleare*	Birds
*P. lophurae*	Birds
Primer sequence	
Isothermo(F) 5′-CGGAAGGGCACCACCAG-3′	
Isothermo(R) 5′-TCACCATCCAAGAAATCAAGAAAG-3′	
Amplicon size: 124 bp	
Probe sequence	
Isoplasmo probe FAM 5′-TCCTACTCTTGTCTTAAACTA-3′ MGB-NFQ	

### Performance testing of primers and probe

**Fig. 1:**
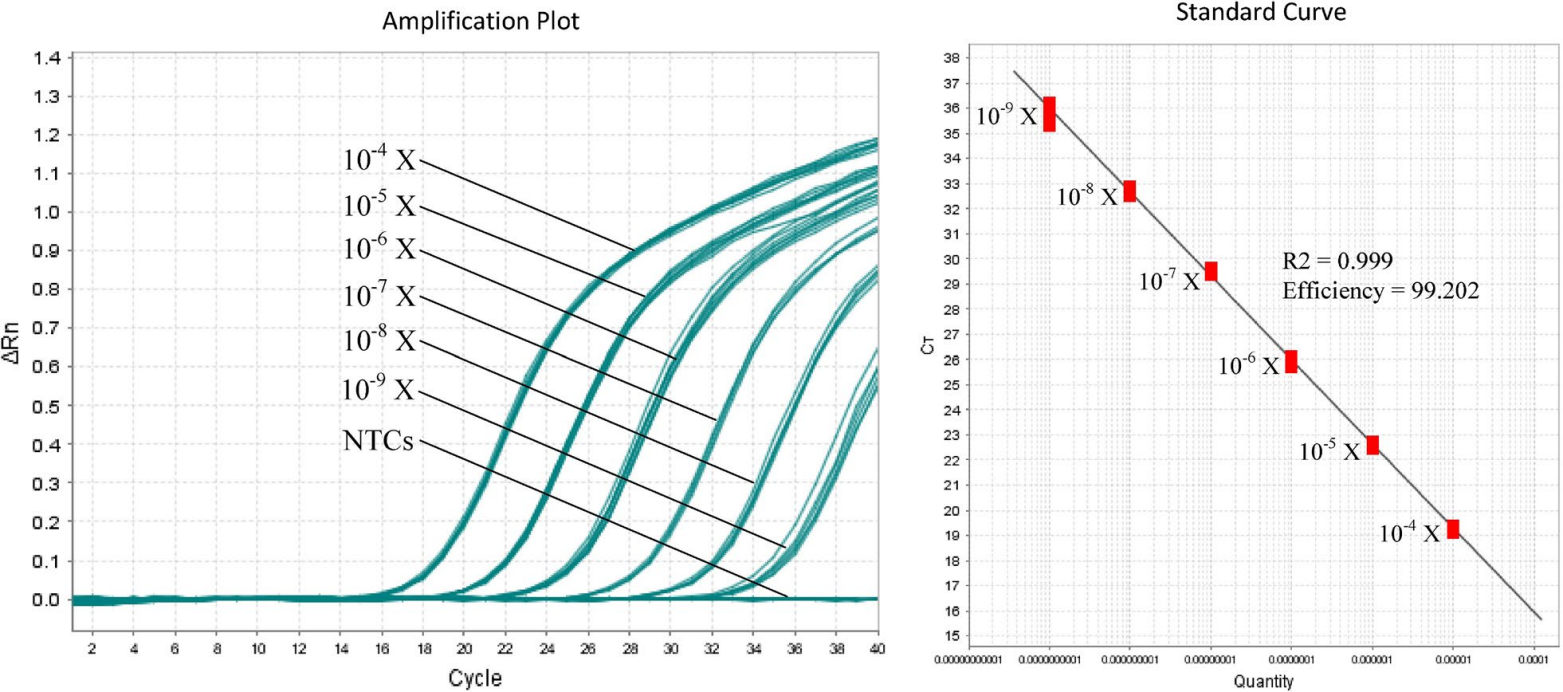
The amplification plot and standard curve of the 10-fold diluted recombinant plasmid DNA samples using qPCR approach. The efficiency of the assay was 99.2 %. The initial concentration of recombinant DNA sample was 50 ng

The qPCR approach demonstrated that Isothermo primers and Isoplasmo probe successfully amplified and detected recombinant plasmid DNA for *P. falciparum*, *P. vivax*, *P. knowlesi*, *P. malariae*, and *P. ovale*, which served as positive controls. No amplification and fluorescent signal was observed for human, bacterial and non-plasmodium parasitic DNA samples. The test was performed in six replicates and the efficiency of the assay achieved 99.2 % (Fig. 1).

### iiPCR method development and evaluation

iiPCR method required higher concentration of primers as compared to qPCR method, which was 100 nM relative to 50 nM. iiPCR approach showed positive detection for the recombinant plasmid DNA for the five human *Plasmodium* spp. by showing + sign on POCKIT™ upon completion of reaction (Table 2). On the other hand, − sign was displayed on the device for no template control (NTC), human DNA and bacterial DNA, whereby their fluorescence ratios after/before reaction were observed to be less than 1.1. Fluorescence ratio of between 1.1 and 1.3 would display ? sign which indicated ambiguous result, while fluorescence ratio of more than 1.3 would show + sign that implied positive detection. The cut-off points for positive, negative and ambiguous results were determined by internal default algorithm of the device.

**Table 2:** Specificity test of iiPCR method

Organism	Fluorescence (550 nm)			Detection
	Before	After	Ratio	
*P. falciparum*	32.111	81.552	2.540	+
*P. knowlesi*	30.803	85.508	2.776	+
*P. vivax*	30.396	80.878	2.661	+
*P. malariae*	30.413	43.614	1.434	+
*P. ovale*	31.760	86.672	2.729	+
*P. coatneyi*	30.640	51.410	1.678	+
*P. cynomolgi*	31.202	46.538	1.492	+
*P. fieldi*	30.574	52.506	1.717	+
*P. inui*	31.002	52.548	1.695	+
Human	32.108	31.869	0.993	−
*A. caviae*	31.688	32.761	1.034	−
*A. aquariorum*	31.288	31.125	0.995	−
*A. hydrophila*	31.648	32.532	0.983	−
*V. parahaemolyticus*	30.125	29.619	1.028	−
*V. harveyi*	30.344	29.875	1.020	−
*S. marcescens*	32.348	32.985	0.985	−
NTC	31.445	32.071	1.020	−
NTC	30.315	29.423	0.971	−
NTC	31.388	30.462	0.971	−
NTC	30.791	31.463	1.022	−
NTC	30.478	30.183	0.990	−

‘+’ positive detection, ‘−’ negative detection, *NTC* no template control

**Fig. 2:**
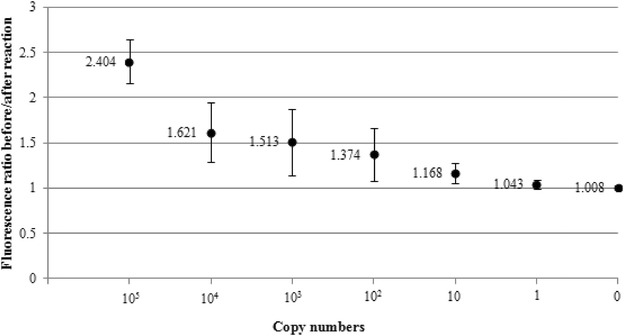
Means and standard deviations of fluorescence ratio after/before reactions for 0–10^5^ copies of recombinant plasmid DNA. Triplicate reactions were performed. Copy numbers of ≥100 with fluorescence ratio of at least 1.374 could be positively detected by POCKIT™

**Fig. 3:**
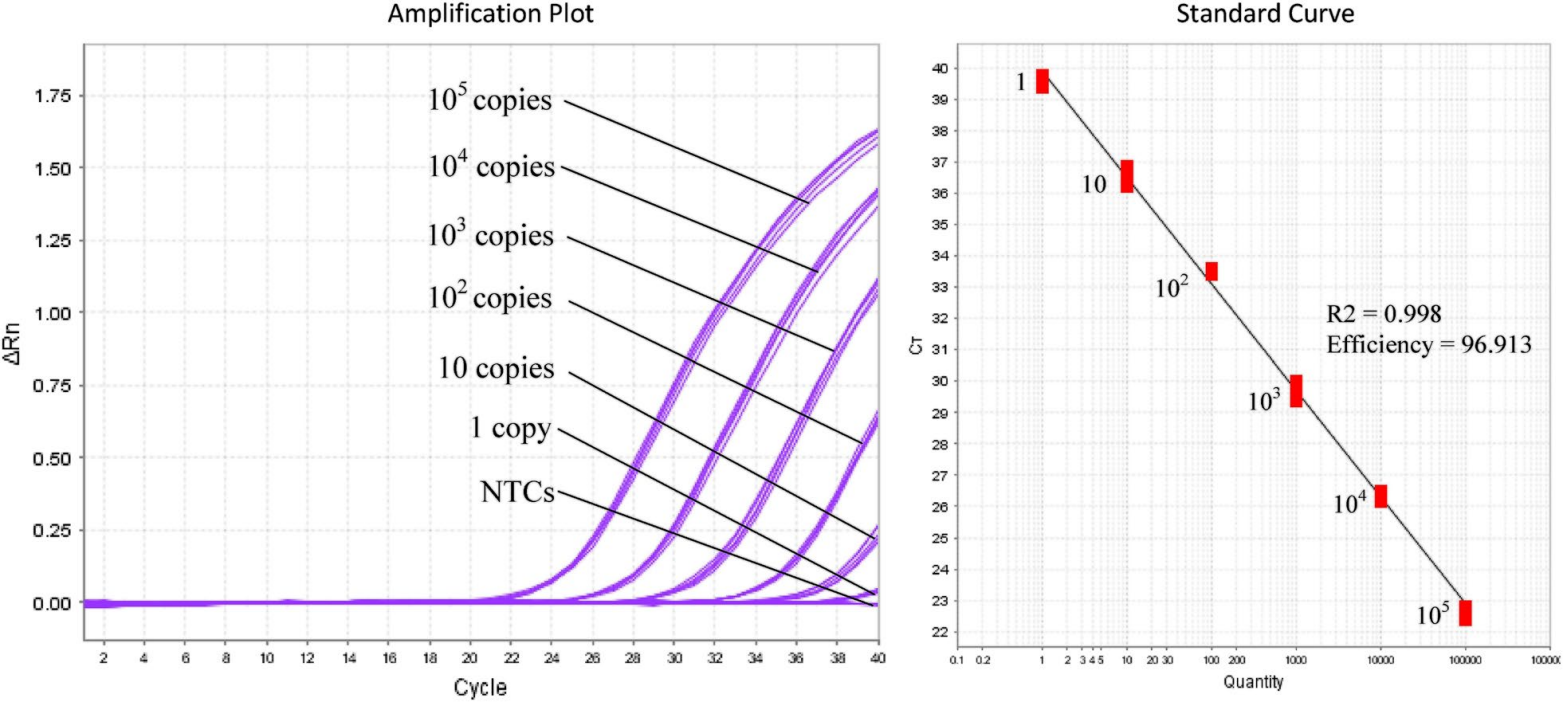
Evaluation of iiPCR assay using qPCR method. The lower detection limit of iiPCR assay 1 copy of recombinant plasmid DNA and the standard curve shows that the assay efficiency is 96.9 %. However the sensitivity and efficiency of the assay are expected to be relatively lower than those of qPCR method when subjected to POCKIT-iiPCR

The lower detection limit of the iiPCR assay was evaluated using a range of 10-fold diluted recombinant plasmid DNA (10^5^–1 copy numbers), and it was found to be ≥100 copy numbers (Fig. 2). The test was performed in triplicates and the means and standard deviations for fluorescence ratios of 10^5^, 10^4^, 10^3^, 10^2^, 10, 1, and 0 copy numbers are shown in Fig. 2. Given that POCKIT™ recognized fluorescence ratio of >1.3 as positive, the 10^2^, 10 and 1 copy number(s) were identified as negative for having that of <1.3. When the same assay was subjected to qPCR method for six replications, detection limit reached 1 copy of recombinant plasmid DNA, whereby 10^5^, 10^4^, 10^3^, 10^2^, 10 and 1 copy numbers produced averaged fluorescent signals at cycle thresholds (Cts) of 22.7, 26.3, 29.7, 33.2, 36.5, and 39.7, respectively (Fig. 3). The efficiency of the iiPCR assay was also examined using qPCR approach and was observed to be 96.9 % (Fig. 3). The fluorescence ratio after/before reaction of the 10-fold diluted recombinant plasmid DNA did not seem to decrease proportionally and this indicated that iiPCR method was not a quantitative method.

### Screening of clinical samples

Among the 55 clinical DNA samples tested, 47 (85.4 %) were detected as +, four (7.3 %) as −, and four (7.3 %) as ? (Table 3). The copy number of plasmodial *18S SSU rRNA* gene in each clinical sample was determined using qPCR approach. Although the lower detection limit of iiPCR assay was previously identified as ≥100 copies of gene, the assay managed to positively detect five clinical samples with 99, 90, 66, 47, and 21 copies of plasmodial *18S SSU rRNA* gene. The four clinical samples having negative results contained zero to ten copies of plasmodial gene. The clinical samples displaying ? results consisted of <100 copies of gene, except for two with 212 and 213 copy numbers, respectively. The fluorescence ratios after/before reaction produced did not correspond to the plasmodial gene copy numbers present in the clinical samples, further suggesting that the iiPCR method is a qualitative or semi-quantitative approach. Negative results with detection displays of − were produced by all the 35 healthy subject samples and the averaged reading for the fluorescence ratios after/before reaction was 0.975, with standard deviation of 0.04.

**Table 3:** Clinical sample screening using iiPCR method

Organism	Fluorescence ratio after/before reaction	Copy number
Detected (47/55)		
PV	1.899	21
PK	1.721	47
PF	2.104	66
PF	1.690	90
PK	1.664	99
PF	1.440	132
PF	1.484	151
PF	1.358	259
PV	1.601	267
PF	1.589	349
PK	1.359	350
PV	1.513	362
PF	1.590	371
PK	1.709	469
PF	1.898	476
PF	2.091	508
PV	1.628	524
PF	1.762	566
PF	2.275	656
PV	1.614	674
PF	1.443	699
PV	1.612	738
PV	2.299	869
PV	1.547	904
PK	1.540	910
PK	1.397	1138
PV	2.095	1141
PF	1.467	1191
PF	1.771	1321
PF	1.606	1457
PF	1.463	1500
PF	1.453	1643
PV	1.619	1683
PF	1.383	1834
PF	1.348	2021
PF	1.555	2102
PF	1.629	3294
PV	1.748	4033
PF	1.572	5261
PK	2.156	6989
PF	2.635	8944
PF	2.115	11,184
PF	2.136	16,977
PK	2.208	18,173
PK	1.927	19,999
PF	1.635	25,365
PF	1.732	30,723
Undetected (4/55)		
PF	1.017	Undetermined
PF	1.109	1
PF	1.013	1
PF	1.003	10
Ambiguous detection (4/55)		
PK	1.2061	38
PF	1.2716	45
PF	1.2891	212
PF	1.2777	213

PF *P. falciparum*, PV *P. vivax,* PK *P. knowlesi*

## Discussion

This study demonstrated the possibility of using portable iiPCR device, POCKIT™, in the detection of *Plasmodium* spp. in clinical samples. Although it may not be as sensitive and quantitative as the qPCR method, it is useful and suitable for malaria detection in the field due to its small size (28 × 25 × 8.5 cm, W × D×H), light weight (2.1 kg), easy operation and direct interpretation of results. Given that this method allows for on-site diagnosis without needing to send samples to a laboratory, it may reduce shipping costs and shorten test turn-around time, which in turn improves disease management [7].

This newly developed malaria diagnostic method, which mainly focused on the detection of the five human *Plasmodium* spp, was the initial and ultimate aim of this study to apply the system in malarial endemic rural areas. It could also be utilized to detect *Plasmodium* spp. infecting monkeys, birds and rodents, which also made it deployable for field surveillance of animal malaria. Specificity test had shown that the assay did not detect organisms other than *Plasmodium*. However, since the assay conferred universal detection of *Plasmodium* spp, it can only serve as a preliminary screening of *Plasmodium* infections in humans or animals. Further confirmation of *Plasmodium* spp. may need to be carried out using another molecular approach. This assay will be ameliorated in the future by permitting it to detect and discern *P. falciparum* from non-*P. falciparum* species using both fluorescence channels (520 and 550 nm) equipped in POCKIT™ device.

The sensitivity test of the iiPCR assay in this study showed that the lower detection limit was ≥100 copies of target DNA sequence, higher than that of qPCR assay which could go down to 1 copy number. Nevertheless, when screening the clinical samples with <100 copies of target DNA, some were found to be positively detected while the rest were either detected as ambiguous or negatively detected. This indicates that the assay could possibly detect samples with lower copy numbers. On the other hand, two samples with 212 and 213 copies of target DNA, which were anticipated to be positively detected, was interpreted as ambiguous. This suggests that iiPCR, which works on the basis of thermal convection, may not be as stable as qPCR, which employs direct heating from the block at desired temperatures. The results also revealed that the iiPCR method was not as quantitative as the qPCR method as the fluorescence ratios did not decline proportionally with the decreased copy numbers of target DNA. Furthermore, the observation that samples with high copy numbers did not consistently obtain high fluorescence ratio and vice versa, once again suggested that the iiPCR assay was qualitative. However, the assay is specific and sensitive enough to detect *Plasmodium* spp in the samples as long as the copy number of the target DNA is ≥ 100, therefore, a second attempt at diagnosis with higher DNA amounts might be needed if ambiguous results are acquired. If translating the copy number into parasite density, whereby a plasmodial genome carries four to eight copies of *18S SSU rRNA* gene [12], the lower detection limit of this method would be 12.5–25 parasites/µl.

The evolution of iiPCR started with the development of Rayleigh-Bénard convection cell by Krishnan and team [3] to perform PCR in a steady convection flow, generated with top and bottom surfaces of the cavity maintained at 97 °C by hot plate and 61 °C by water cooled top plate, respectively. After that, Chou et al. [4] modified the method by using dry bath to heat the bottom of a capillary tube at a fixed temperature of 95 °C, followed by DNA amplification which is rendered by temperature gradient created when the tube is cooled by the surrounding air. But since fluctuations of ambient temperature, especially at temperatures >38 °C would affect the performance of the reaction, Chang and team [13] established the first insulated isothermal PCR (iiPCR) by adding thermal baffles which are able to stabilize the temperature gradient inside the reaction tube. Gel electrophoresis was used as the DNA product detection method until the fluorescent hydrolysis probe (Taqman™ probe) technology and optical detection module, which enables fluorescent signal detection, were integrated into the iiPCR system [5]. This further modified system was reported to be able to offer low-cost and rapid on-site pathogen detection, with a high sensitivity (100 %) and specificity (96.67 %) in the case of white spot syndrome virus detection [5]. Today, the system is commercially available with all-in-one functions allowing for iiPCR/reverse transcription, signal detection and data interpretation in one device.

Currently, the simplest way to diagnose malaria is using the rapid diagnostic test (RDT) and its use has increased over the past few years, especially in the developing countries. It is a test strip either in a plastic cassette enclosure or attached to cardboard that detects malaria antigen in a small amount of blood (5–15 µl) and results can be obtained in 5–20 min [14]. Among many commercial tests that have been developed and tested in various countries, only one product received FDA clearance in June 2007 [14–20]. RDTs available on the market can detect *P. falciparum* alone, *P. falciparum* and *P. vivax*, as well *P. falciparum* from non-*P. falciparum* species [15–20]. Overall, RDTs for *P. falciparum* might attain greater than 95 % sensitivity but parasite density needs to be ≥500/µl of blood [14]. Nevertheless, the product testing programme organized by WHO and other partners set an even lower parasite density of 200/µl (which is below the mean parasite density found in many populations with endemic malaria) as their evaluation standard of RDT in order to assure the product is reliable to identify clinical malaria in various settings [21, 22]. False-positive and false-negative results are common for RDT as it is affected by cross-reactivity with rheumatoid factor [23, 24] and the presence of inhibitor in blood [25], as well as temperature and humidity of endemic areas [14, 26].

Of late, loop-mediated isothermal amplification (LAMP), developed by Notomi et al. [27], emerged as another, similar isothermal DNA amplification method and has gained popularity as many researchers have reported it to be rapid, accurate, cost-effective, and suitable for on-site surveillance and diagnosis. The set-up of LAMP is relatively simpler and cheaper than POCKIT-iiPCR as no specific device, only water/dry bath, and no specific tubes, only normal microcentrifuge tubes, are required to perform the amplification. LAMP is also claimed to be able to amplify a few copies of DNA to a vast amount of end product, in which turbidity is observable with the naked eye within an hour. The reagents used are also much cheaper than those used for POCKIT-iiPCR. Although multiplexing cannot be done for LAMP method as compared to POCKIT-iiPCR, which permits duplexing, LAMP can be performed in high-throughput even with single-plexing in separate tubes because it only uses water/dry bath for heating and thus the capacity of the water/dry bath is much more dependable. Its resistance to inhibitory substances present in biological samples allows simpler sample preparation [28], such as heating of blood samples [29], which in turn saves time and cost in the sample processing steps required for PCR and POCKIT-iiPCR. This method has been widely developed for malaria detection as it can be performed in resource-limited settings, and a LAMP kit for malaria detection, namely Loopamp™ MALARIA Pan/Pf detection Kit, has now been brought to the market [30, 31]. LAMP method developed by Han and team has achieved the detection of up to four species of *Plasmodium*, with detection limit of ten copies of the target *18S rRNA* genes for *P. malariae* and *P. ovale*, and 100 copies for *P. falciparum* and *P. vivax* [32].

However, the tedious part of the LAMP method development is the primer design, as four or six primers should be included in a reaction. The method developed by Han and team involved a total of 30 primers, six primers in each primer set for genus *Plasmodium* and four *Plasmodium* species [32]. Although it has been said that the recognition of six or eight distinct regions on target sequence by the primers may increase the specificity of the method, it will also increase the likelihood of primer-primer interactions, as well as primer design constraints. The major shortcoming of LAMP is false-positive results resulting from carry-over contamination of amplicons and non-specific amplification of non-target sequence [33], which are ruinous for a diagnostic method. The elimination of post-amplification procedures and the coupling of sequence-specific Taqman™ probes, as well as the use of uracil-DNA glycosylase (UDG) contained master mix, in POCKIT-iiPCR can preclude both issues. Multiplexing in a reaction is not applicable with LAMP method because *Bst* polymerase, which lacks 5′-3′ exonuclease activity, does not support the use of hydrolysis probes, although Tanner and his group developed a multiplexed real-time LAMP which allowed detection of one to four targets simultaneously [34]; the fluorescent signal detection is another problem to be solved. On the other hand, with the all-in-one function of POCKIT, fluorescence signal emitted by FAM™- and VIC^®^-labelled probes during iiPCR can be detected directly by the built-in detector in the device.

## Conclusions

The POCKIT-iiPCR assay developed in this study provides another option besides RDT and LAMP in the diagnosis of malaria. Despite the slightly higher cost (approximately US$2 per sample) and longer result generation time, the high specificity and low detection limit are some of the advantages of POCKIT-iiPCR assay over RDT (US$0.15–1.50 per sample) [14]. A POCKIT nucleic acid analyser costs approximately US$7000, which might be worth investing in a point-of-care PCR platform to be used in the field. POCKIT-iiPCR does not require separate equipment to perform PCR and detection, and most importantly, it does not have carry-over contamination and false-positive result issues, as occurs in the LAMP method. The design of primers and probe is relatively simpler than the LAMP method, although the cost is unfortunately higher than that of LAMP, as probe is utilized and final volume of a reaction is larger (50 µl compared to 25 µl in LAMP). The use of probes expands the potential of POCKIT-iiPCR to perform multiplexed detection in a reaction, which in turn can save time and reagents for a diagnosis. Electricity is required to operate the device but a normal generator is sufficient enough as the device’s power consumption is low. DNA extraction step cannot be omitted in this method at the moment, however direct loading of blood samples will be attempted in future by modifying the master mix in order to withstand inhibitors present in the blood yet allowing iiPCR and fluorescent signal emission to occur. Another limitation of this study was the unavailability of plasmodial cultures to evaluate the assay sensitivity. Overall, the iiPCR assay can be refined by enabling it to detect *P. falciparum* and other species or distinguish it from non-*P. falciparum* species.
